# Quality of Survey Responses at Older Ages Predicts Cognitive Decline and Mortality Risk

**DOI:** 10.1093/geroni/igac027

**Published:** 2022-04-20

**Authors:** Stefan Schneider, Doerte U Junghaenel, Erik Meijer, Elizabeth M Zelinski, Haomiao Jin, Pey-Jiuan Lee, Arthur A Stone

**Affiliations:** Center for Self-Report Science, and Center for Economic and Social Research, University of Southern California, Los Angeles, California, USA; Department of Psychology, University of Southern California, Los Angeles, California, USA; Center for Self-Report Science, and Center for Economic and Social Research, University of Southern California, Los Angeles, California, USA; Department of Psychology, University of Southern California, Los Angeles, California, USA; Center for Economic and Social Research, University of Southern California, Los Angeles, California,USA; Leonard Davis School of Gerontology, University of Southern California, Los Angeles, California,USA; Center for Economic and Social Research, University of Southern California, Los Angeles, California,USA; School of Health Sciences, University of Surrey, Guildford, UK; Suzanne Dworak-Peck School of Social Work, University of Southern California, Los Angeles, California, USA; Center for Self-Report Science, and Center for Economic and Social Research, University of Southern California, Los Angeles, California, USA; Department of Psychology, University of Southern California, Los Angeles, California, USA

**Keywords:** Aging, Careless responding, Cognitive ability, Self-report, Survey satisficing

## Abstract

**Background and Objectives:**

It is widely recognized that survey satisficing, inattentive, or careless responding in questionnaires reduce the quality of self-report data. In this study, we propose that such low-quality responding (LQR) can carry substantive meaning at older ages. Completing questionnaires is a cognitively demanding task and LQR among older adults may reflect early signals of cognitive deficits and pathological aging. We hypothesized that older people displaying greater LQR would show faster cognitive decline and greater mortality risk.

**Research Design and Methods:**

We analyzed data from 9, 288 adults 65 years or older in the Health and Retirement Study. Indicators of LQR were derived from participants’ response patterns in 102 psychosocial questionnaire items administered in 2006–2008. Latent growth models examined whether LQR predicted initial status and change in cognitive functioning, assessed with the modified Telephone Interview for Cognitive Status, over the subsequent 10 years. Discrete-time survival models examined whether LQR was associated with mortality risk over the 10 years. We also examined evidence for indirect (mediated) effects in which LQR predicts mortality via cognitive trajectories.

**Results:**

After adjusting for age, gender, race, marital status, education, health conditions, smoking status, physical activity, and depressive symptoms, greater LQR was cross-sectionally associated with poorer cognitive functioning, and prospectively associated with faster cognitive decline over the follow-up period. Furthermore, greater LQR was associated with increased mortality risk during follow-up, and this effect was partially accounted for by the associations between LQR and cognitive functioning.

**Discussion and Implications:**

Self-report questionnaires are not formally designed as cognitive tasks, but this study shows that LQR indicators derived from self-report measures provide objective, performance-based information about individuals’ cognitive functioning and survival. Self-report surveys are ubiquitous in social science, and indicators of LQR may be of broad relevance as predictors of cognitive and health trajectories in older people.

**Translational Significance:** Identifying early indicators of cognitive decline at older ages is of paramount importance in our aging society. Low-quality responding (LQR) in self-report surveys has been associated with lower cognitive abilities, but whether LQR can predict cognitive decline and mortality risk has been understudied. In a longitudinal U.S. nationally representative study, we found that people who showed more LQR had concurrently worse cognitive test scores, more rapid cognitive decline, and they were more likely to die over up to 10 years of follow-up. Examining LQR in survey responses may facilitate the development of new strategies for early detection of pathological aging.

Accurately responding to self-report questionnaires requires a great deal of cognitive effort. For each question, respondents are expected to identify its meaning, thoroughly search all relevant information from memory, integrate the retrieved information, and map their final judgment onto one of the provided response options (Schwarz & Knäuper, 2012; Tourangeau et al., 2000). A widely recognized concern in psychological science is that some participants are not willing or able to adequately perform these mental steps and therefore fail to give precise and plausible answers. Such response behaviors have been variously described as careless or inattentive responding (Meade & Craig, 2012), insufficient effort responding (Huang et al., 2015), survey satisficing (Krosnick, 1991), protocol invalidity (Johnson, 2005), aberrant responding (Conijn et al., 2015), or, more neutrally, low-quality responding (LQR; Buchanan & Scofield, 2018; Chen, 2011). We prefer the relatively neutral term “low-quality responding” in this article over careless or insufficient effort responding to avoid a connotation that the resultant data are exclusively due to respondent unwillingness. A sizeable body of research has documented that LQR can undermine the accuracy and precision of study results, and has recommended excluding participants with LQR from data analysis to mitigate biases in empirical research (e.g., Huang et al., 2015; Johnson, 2005; Meade & Craig, 2012; Schneider et al., 2018).

In the present study, we take the much less frequently considered perspective that LQR can also carry substantive meaning. From this perspective, LQR may reflect suboptimal information processing and potential cognitive functioning limitations. Various authors have suggested that cognitive deficits may limit the ability to accurately complete self-report questionnaires. For example, several studies have documented that older adults with impaired cognitive functioning show higher rates of item nonresponse in questionnaires, with more missing values due to skipped questions or selection of “do not know” answers (Colsher & Wallace, 1989; Fuchs, 2009; Knäuper et al., 1997; Kutschar et al., 2019). In a population-based survey of over 3,000 respondents 65 years and older, Colsher and Wallace (1989) found that respondents who had poorer physical health and worse cognitive functioning were more likely to give internally inconsistent (intraindividually unreliable) responses. Lower cognitive abilities at older ages have also been associated with a greater likelihood of acquiescent responding, that is, agreeing with survey items regardless of question content (Lechner & Rammstedt, 2015). Each of these behaviors is reflective of LQR in that they do not result from intentional deception (such as trying to make a favorable impression), but are types of invalid, unreliable, or erroneous responding.

Although these studies suggest relationships between LQR and cognitive deficits, they were cross-sectional in nature and did not address the question whether LQR may be indicative of longitudinal functioning trajectories. In a previous study, we found that several measures of LQR were predictive of subsequent dementia onset in a population-representative sample of middle-aged and older adults (Schneider et al., 2021). The present longitudinal study builds on this prior work by examining whether LQR is an early indicator of cognitive decline and mortality risk in late adulthood. The rationale for this is that inaccuracies and imprecisions when performing cognitively demanding tasks represent some of the earliest signals of neurodegenerative changes at the end of life (Sperling et al., 2011). Thus, LQR among older adults may reflect an early behavioral manifestation of reduced cognitive abilities and general neurobiological disturbance associated with degenerating brain functions. Inconsistencies in cognitive test performance (e.g., reaction time variability) are well-known early predictors of general cognitive decline (Lu et al., 2020) and mortality (MacDonald et al., 2008). Accordingly, even though self-report questionnaires are not formally designed as cognitive tasks, erroneous, inconsistent, or implausible responses in surveys may represent predictors of pathological aging, including cognitive changes that are associated with increased risk of mortality.

We pursued three aims in this research. First, to expand beyond previous findings that indicators of LQR are cross-sectionally associated with lower cognitive abilities in older adults, we investigated whether they prospectively predicted cognitive decline over up to 10 years of follow-up in a U.S. nationally representative cohort of adults aged 65 and older. We hypothesized that LQR would be associated with more negative cognitive trajectories, that is, concurrently worse cognitive functioning and steeper cognitive decline thereafter, independent of age. Second, we investigated whether LQR was associated with mortality risk. We hypothesized that LQR would predict a greater risk of mortality over the follow-up period. Third, consistent with the idea that LQR could be an early indicator of a cognitive trajectory preceding impending death, we examined evidence for indirect (mediated) effects in which LQR predicts mortality via more negative cognitive trajectories. Following recommendations, we considered relevant covariates (sociodemographic factors, health conditions, and health behaviors) that might confound direct effects or indirect (mediated) effects (VanderWeele, 2019).

## Research Design and Methods

### Sample

The analytical sample was drawn from the Health and Retirement Study (HRS), a panel study that has been collecting data on social, health, and economic issues in a sample of older Americans since 1992 (http://hrsonline.isr.umich.edu/). HRS participants are interviewed every 2 years. Initial respondents consisted of individuals between 51 and 61 years of age and their spouses, and new cohorts were added over time to render the sample representative of the U.S. population 50 years and older (Sonnega et al., 2014).

At the 2006 and 2008 waves, the HRS started administering a package of psychosocial self-report measures referred to as a leave-behind survey (Smith et al., 2013). This self-administered questionnaire package was given to respondents at the end of their face-to-face interview, to be returned in the mail. A random 50% of the panel members were first selected to complete the questionnaires in 2006 (the response rate was 90% among those selected), and the remaining 50% were asked to complete it in 2008 (with a response rate of 89%; Smith et al., 2013); respective data from these waves were considered the baseline for the present analyses. We included all participants who were 65 years of age or older at baseline and who completed the leave-behind questionnaires by themselves. Of 14,331 individuals who responded, we excluded *N* = 4,876 individuals who were between 51 and 64 years of age because the HRS did not administer some cognitive tests to these participants. We also excluded *N* = 167 individuals whose questionnaires were completed by proxy respondents. The resulting analytic sample size was *N* = 9,288. Compared with the analysis sample, those 65 years or older who did not return or self-complete the leave-behind questionnaires were on average 3.14 years older, were 10.75% less likely White, were 12.10% less likely married, had 1.32 fewer years of education, and were 23.59% more likely to die during the follow-up period. All participants provided informed consent as part of the HRS, and the research was approved by the relevant Institutional Review Boards.

### Measurement

#### Indicators of low-quality responding

We analyzed responses to 102 self-report questions included in 21 multi-item rating scales in the HRS leave-behind survey (between 3 and 7 items per scale, mode = 5 items) to derive several indicators of LQR. The leave-behind survey was administered to assess a range of psychosocial constructs (e.g., optimism, hopelessness, the Big Five personality traits). For details, see Supplementary Material, as well as Smith et al. (2013). We only analyzed responses to scales that were administered both in 2006 and 2008 (i.e., the baseline for the analyses) and that were applicable to all respondents (i.e., questionnaires on participants’ experiences with their spouse, children, or work environment that are relevant only to respondent subgroups were excluded).

Given that there is no single best way to identify LQR, we computed multiple indicators that address a range of different response patterns, consistent with recommendations (Curran, 2016; Meade & Craig, 2012). These indicators have been discussed in detail elsewhere (Colsher & Wallace, 1989; Curran, 2016; Lechner & Rammstedt, 2015; Meade & Craig, 2012; Schneider, 2018); detailed information is given in the Supplemental Material. We derived (a) an index of individual *response inconsistency*, calculated as the residual item-level variability around a person’s scale scores, (b) a *multivariate outlier* index, calculated as the Mahalanobis distance of a person’s responses across all items, (c) an item response theory (IRT)-based index of *misfitting* item response patterns, calculated as the average *l*_*z*_ person-fit statistic across scales, (d) an index of *acquiescent response bias*, derived from a nominal IRT model that estimates the tendency to agree with statements regardless of item content, and (e) an *item nonresponse* index, calculated as the proportion of questions with skipped responses.

We created an overall summary index of LQR by computing the standardized average of the five different LQR indicators. This summary index had adequate internal consistency reliability (alpha = .81).

#### Cognitive assessment

Cognitive functioning was assessed with seven cognitive tasks the HRS administers every 2 years based on a modified version of the Telephone Interview of Cognitive Status (Ofstedal et al., 2005). The measure taps performance on immediate and delayed free recall, working memory and mental processing (serial 7s), and mental status measures (backward counting, object naming, president/vice president naming, time orientation). We obtained the overall sum score of the cognitive tasks (total possible score range: 0–35) from the publicly available version of the HRS cognitive measures, in which missing scores on individual cognitive tasks were imputed (McCammon et al., 2019). Cronbach alphas ranged from .71 to .76 across waves. Cognitive scores for each of the 5 assessment waves (10 years) following the baseline assessment were used in the analyses.

#### Mortality

Mortality data were derived from the HRS exit interviews, which are conducted with a proxy-respondent after a participant’s death. The proxy respondents are identified from the deceased’s social network, most commonly the spouse or a close family member. The HRS keeps frequent contact with study participants. As a consequence, exit interviews are completed for almost all (>95%) deceased individuals, providing near complete mortality surveillance (Weir, 2016). Because our analyses used a discrete-time survival model, we retained only year (not day or month) of death from the HRS mortality data. Year of death was coded in years since the baseline interview, that is, coded as 1 if a person died within 12 months after the baseline interview, as 2 if a person died between more than 12 up to 24 months of the interview, and so on. Deaths were coded up until 10 years following the baseline assessment, at which point the study was right censored.

#### Covariates

We included the demographic covariates age, gender (women or men; reference category was women), race (White or other race, reference category was White), marital status (married or not, reference was married), and years of education (continuous variable). Physical health covariates related to mortality risk included the presence of chronic health conditions hypertension, diabetes, heart disease, and stroke (none or ≥1 condition, reference was none), smoking (never smoked, former smoker, or smokes now; reference was never smoked), and exercise (less than once per month, 1–4 times per month, more than once per week; reference was less than once per month). All categorical covariates were dummy-coded, education was centered at 12 years, and age was centered at 65 years. We also controlled for depression at baseline to test whether this could account for the effects of LQR, given that depression has been associated with increased risk of cognitive decline (Wilson et al., 2004), mortality (Raykov et al., 2017), and LQR (Conijn et al., 2020). Depressive symptoms were measured with the HRS eight-item version of the Center for Epidemiologic Studies—Despresion scale (CES-D; Radloff, 1977). Cronbach’s alpha was .80 in the present sample. Assessment of all covariates took place during HRS interviews conducted before participants were given the leave-behind survey.

### Statistical Analyses

Preliminary analyses examined associations between the index of LQR and the covariates assessed at baseline, using Pearson correlations for continuous covariates and using *t*-tests and analyses of variance for categorical covariates.

**Figure 1.**
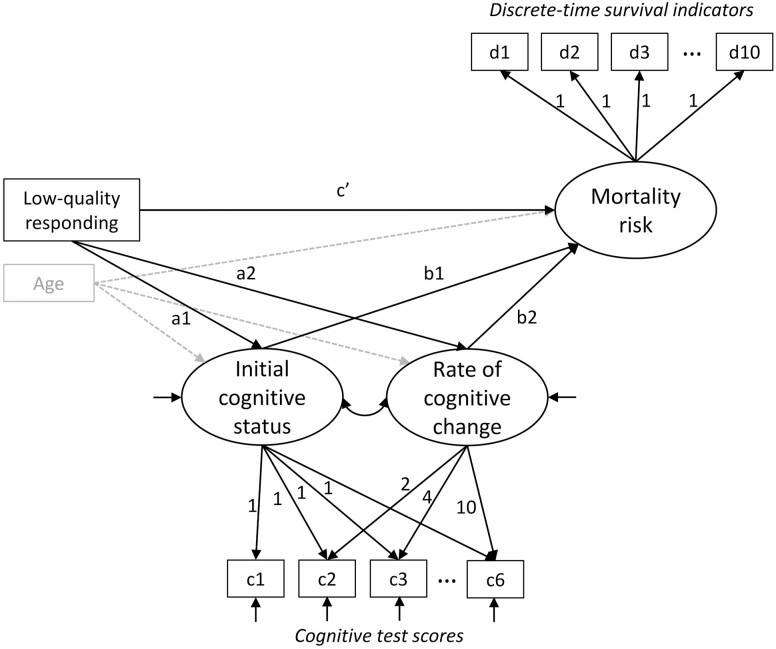
Joint latent growth model of cognitive functioning (initial cognitive status and rate of cognitive change) and discrete-time survival model (mortality risk), including the predictor variable (low-quality responding) and covariate age. Additional covariates included in the models are not shown for visual clarity. c1–c6 are observed continuous test scores for each assessment wave, and they are used as indicators of intercept (initial status) and linear change (rate of change) factors in a latent growth model. d1–d10 are coded as 0 if the respondent is still alive, 1 if the respondent died during that year, and missing in subsequent years following death; they serve as indicators of a mortality risk factor with loadings constrained at 1 following the assumption of proportional hazard odds.

The primary analyses were performed using latent variable models in M*plus* version 8.7 (Muthén & Muthén, 2017). Latent variable modeling provided a flexible framework to examine predictors of longitudinal cognitive trajectories (Hypothesis 1) and mortality risk (Hypothesis 2), and to combine these two components into a joint model testing indirect effects (Hypothesis 3) (Muthén, 2002; Wang et al., 2012). The combined model is illustrated in Figure 1, and we describe the model components below.

#### Latent growth model

To examine predictors of longitudinal cognitive trajectories, we used latent growth models (Singer & Willett, 2003) in which respondents’ initial (i.e., baseline) cognitive status and rate of cognitive change were expressed as latent variables estimated from the six repeated cognitive test scores (c1–c6 in Figure 1) collected over the 10-year period. The latent initial status and change variables served as outcomes and were regressed on LQR and covariates. Both linear and curvilinear (quadratic) models of change were initially compared in preliminary analyses; the more parsimonious linear growth model was retained because it fit the data well (χ ^2^ goodness of fit = 135.8 [*df* = 16, *p* < .01], comparative fit index = .995, root mean square error of approximation = .029 with 95% confidence interval = 0.025–0.034) and because there was no significant variance in the quadratic growth factor (*p* = .19).

#### Discrete-time survival model

We predicted mortality risk using a discrete-time survival model (Raykov et al., 2017; Singer & Willett, 2003) estimated from the 10 years of mortality data following baseline (d1–d10 in Figure 1). Each year was coded 0 if the respondent was still alive, 1 if the respondent had died during that year, and missing in years following death, where an underlying mortality risk factor describes the latent propensity of an individual dying in a particular year provided that the individual has not died before that year. The so-called proportional hazard odds (PHO) model assumes that the effect (hazard odds ratio [OR]) of all predictors (LQR and covariates) on mortality risk is constant across years (as indicated by loadings to the mortality risk factor constrained at 1.0 in Figure 1). We tested this PHO assumption by comparing the Bayesian information criterion (BIC, where lower values indicate better model fit) of models that assumed time-constant effects versus allowed for time-varying effects across years, for all predictors simultaneously (Raykov et al., 2017). The BIC of the former model was considerably lower (BIC = 26,072.42) compared to the latter model (BIC = 26,877.81), supporting the use of the PHO model.

#### Indirect (mediated) effect model

Evidence for indirect effects of LQR on mortality risk via cognitive trajectories was tested by combining the latent growth and discrete-time survival models into a joint mediation model (Wang et al., 2012). We used the product of coefficients method to estimate specific indirect effects of LQR on mortality risk via participants’ initial cognitive status (multiplied a_1_b_1_ paths in Figure 1) and via cognitive change (a_2_b_2_ paths; Tein & MacKinnon, 2003). We used bootstrapping with 1,000 bootstrap draws to estimate 99% confidence intervals (CIs) of the indirect effects.

Our primary analyses are based on the overall summary index of LQR. Secondary analyses were conducted for each of the five individual LQR indicator variables to evaluate the robustness of the results across different indicators.

In all analyses, we initially adjusted the effect of LQR for age (Model 1) and then adjusted for sociodemographic covariates age, gender, race, marital status, education (Model 2). Model 3 additionally adjusted for physical health variables (health conditions, smoking status, physical activity) that could be on the path of a relationship between LQR and mortality, and Model 4 included depressive symptoms. Missing values on covariates (median = 0.1%, range = 0%–2.0% missing across covariates) were imputed using five multiple imputations. Because the results of latent growth models can be biased if missing values on the cognitive scores due to participant death are not taken into account, we report effects for longitudinal cognitive trajectories estimated from the combined model with survival as distal outcome; this appropriately adjusts the growth model estimates for informative censoring due to participant death (Wang et al., 2012). All analyses were conducted using maximum likelihood parameter estimation. Because multiple tests were conducted, we considered results at a level of *p* < .01 statistically significant to control for inflation of Type I error due to multiple comparisons; accordingly, we report 99% CIs.

## Results

### Preliminary Analyses

The average age at baseline was 75.0 years (range = 65–104 years). Participants had an average of 12.3 years of education, 57.8% were women, and 84.9% were White. About three quarters of respondents reported a chronic health condition, about two fifths had never smoked cigarettes, and one third exercised at least once per month (see Table 1).

**Table 1.** Sample Characteristics and Associations With the Summary Index of Low-Quality Responding

Characteristic	Mean (*SD*)	%	Correlation with LQR	Group mean (*SD*) LQR *z*-score
Age	75.00 (7.09)		.12***	
Years of education	12.28 (3.14)		−.31***	
Gender				*t*_(1,9286)_ = 6.92***
Male		42.16%		−.08 (0.98)
Female		57.84%		.06 (1.01)
Race				*t*_(1,9286)_ = 25.51***
White		84.88%		−.11 (0.94)
Other		15.12%		.61 (1.08)
Marital status				*t*_(1,9286)_ = −20.19***
Not married		41.36%		.24 (1.03)
Married		58.64%		−.17 (0.94)
Chronic health condition				*t*_(1,9239)_ = 5.83***
No		24.75%		−0.11 (0.98)
Yes		75.25%		0.03 (1.00)
Smoking				*F*_(2,9217)_ = 9.65***
Never		42.91%		−.00 (1.01)
Former		47.56%		−.03 (0.99)
Current		9.53%		.14 (1.02)
Exercise				*F*_(2,9275)_ = 102.86***
<1 time/month		66.41%		.10 (1.02)
1–4 times/month		12.33%		−.17 (1.00)
>once/week		21.26%		−.23 (0.88)
CES-D score	1.37 (1.87)		.27***	

*Notes*: CES-D = Center for Epidemiologic Studies—Depression scale. LQR = low-quality responding. Chronic health conditions included hypertension, diabetes, heart disease, and stroke.

***p < .001.

Higher scores on the summary index of LQR showed significant positive associations with older age, female gender, having a chronic health condition, current smoking, and higher depression levels. In addition, higher LQR scores were significantly negatively associated with more years of education, being White, married, and more frequent exercise (Table 1).

We inspected the correlation between the summary LQR and cognitive functioning scores obtained at the baseline assessment. Higher LQR scores were significantly negatively correlated with baseline cognitive functioning scores, *r* = −.35. The magnitude of this correlation was similar to the correlation between baseline age and cognitive functioning, *r* = −.30.

### LQR and Cognitive Trajectories

In latent growth models, we found that higher summary LQR scores were significantly associated with lower initial cognitive status after adjusting for age (Model 1). A 1-*SD* higher LQR was associated with a 1.55-point lower initial cognitive status on a 0–35 scale, a standardized effect size of −0.37 *SD*s. The effect became less pronounced after adjusting for additional demographic characteristics (Model 2), physical health variables (Model 3), and depression (Model 4), but it remained statistically significant in the model with all covariates. The standardized effect of LQR on participants’ initial cognitive status in the final Model 4 was −0.17 *SD*s (Table 2).

**Table 2.** Regression Coefficients for Low-Quality Responding as Predictor of Initial Cognitive Status and Rates of Cognitive Change in Latent Growth Models

Regression coefficients	Model 1	Model 2	Model 3	Model 4
Unstandardized coefficients				
Effect on initial cognitive status	−1.55 [−1.66, −1.43]	−0.82 [−0.93, −0.71]	−0.80 [−0.91, −0.69]	−0.71 [−0.83, −0.60]
Effect on yearly rate of cognitive change	−0.04 [−0.06, −0.03]	−0.04 [−0.05, −0.02]	−0.03 [−0.05, −0.02]	−0.03 [−0.05, −0.02]
Standardized coefficients				
Effect on initial cognitive status	−0.37 [−0.39, −0.34]	−0.20 [−0.22, −0.17]	−0.19 [−0.22, −0.16]	−0.17 [−0.20, −0.14]
Effect on yearly rate of cognitive change	−0.11 [−0.16, −0.07]	−0.09 [−0.14, −0.05]	−0.09 [−0.14, −0.04]	−0.08 [−0.13, −0.03]

*Notes*: Values in square brackets are 99% confidence intervals.

Higher LQR scores at baseline were also prospectively associated with more negative rates of cognitive change (Table 2). Adjusting for age (Model 1), a 1-*SD* higher LQR was associated with a 0.04 point greater cognitive decline per year, a 23.5% greater decline than the model-estimated average cognitive decline of 0.17 points per year (the standardized effect was −.11 *SD*s). This association was slightly attenuated but remained significant when additional demographic characteristics were controlled (Model 2), and it remained unchanged when health variables and depression scores were added (Models 3 and 4). The standardized effect of LQR on cognitive change in the final Model 4 was −0.08 *SD*s (Table 2).

### LQR and Mortality

**Figure 2.**
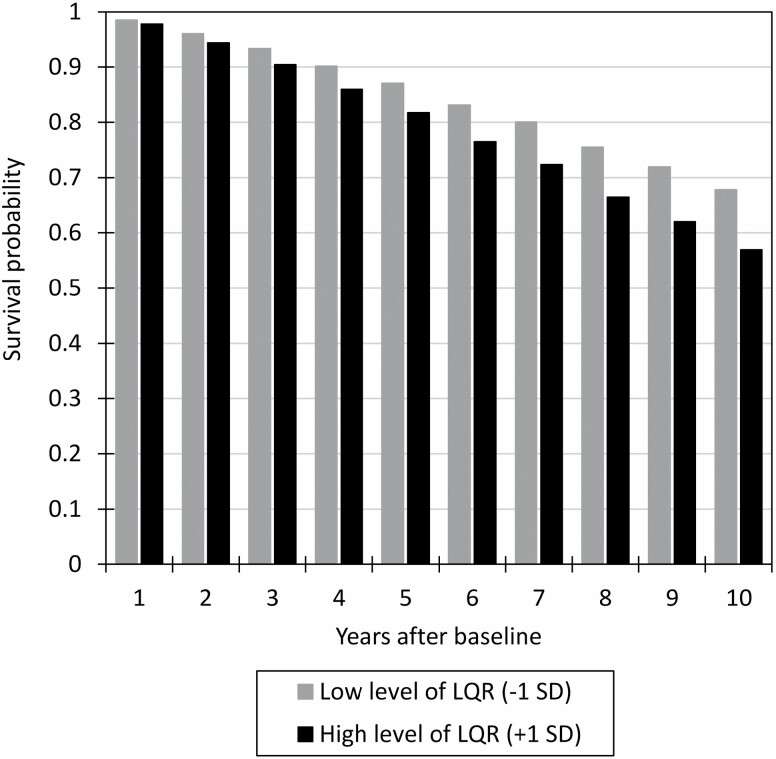
Model 1 results from the discrete-time survival analyses. Estimated survival probabilities are shown for individuals with lower (mean − 1 *SD*) and higher (mean + 1 *SD*) levels of low-quality responding at baseline, controlling for baseline age. LQR = low-quality responding.

Over the 10 years of follow-up, 3,713 (39.98%) of the participants died. In discrete-time survival models, we found that higher summary LQR scores were significantly associated with greater mortality risk. After adjusting for baseline age (Model 1), a 1-*SD* higher LQR was associated with a higher likelihood of death in a given year (hazard OR = 1.21; 99% CI = 1.17, 1.25). Figure 2 shows the estimated survival probabilities for respondents with low (mean − 1 *SD*) and high (mean + 1 *SD*) levels of LQR over the 10 years of follow-up. The predicted survival rates 5 years after baseline were 87.0% versus 81.8% for respondents with low versus high LQR, and the survival rates 10 years after baseline were 67.8% versus 56.9% for respondents with low versus high LQR levels, respectively.

The association between baseline LQR and mortality risk remained significant after controlling for additional demographic characteristics (Model 2, hazard OR = 1.19 [99% CI = 1.15, 1.23]), physical health variables (Model 3, hazard OR = 1.17 [99% CI = 1.13, 1.21]), and baseline depression levels (Model 4, hazard OR = 1.14 [99% CI = 1.10, 1.18]). In the final model, the predicted survival rates 10 years after baseline were 66.3% versus 58.8% for respondents with low versus high LQR levels, respectively.

### Indirect Effects of LQR on Mortality Risk via Cognitive Trajectories

Before investigating evidence for indirect (mediated) effects, we examined the effects of participants’ cognitive trajectories on mortality in models that did not include predictor variables. We found that a 1-*SD* (4.20 points) lower initial cognitive status was associated with a significantly higher mortality risk (hazard OR = 1.46; 99% CI = 1.37, 1.54). A 1-*SD* (0.36 points) more negative rate of cognitive change was also associated with a significantly higher mortality risk (hazard OR = 1.84; 99% CI = 1.66, 2.03).

Adjusting for age (Model 1), we found that participants’ initial cognitive status and rate of cognitive change both partially explained the association between LQR and mortality. A 1-*SD* higher LQR was associated with a significantly higher mortality risk by a hazard OR of 1.11 (99% CI = 1.08, 1.14) via a lower initial cognitive status and by a hazard OR of 1.05 (99% CI = 1.03, 1.08) via a more negative rate of cognitive change. The combined indirect effect explained 78.0% of the total effect of LQR on mortality risk, whereby the specific indirect effects via initial cognitive status and via cognitive change explained 52.2% and 25.9% of the total effect, respectively. Both of these indirect effects remained significant after controlling for demographic characteristics, health variables, and depression scores (Models 2–4, Table 3). In the final model, the combined indirect effect explained 64.3% of the total effect of LQR on mortality risk, with the specific indirect effects via initial cognitive status and cognitive change explaining 35.0% and 29.4% of the total effect, respectively.

**Table 3.** Hazard Ratios for Total, Indirect, and Direct Effects of Low-Quality Responding on Mortality

Model Parameters	Model 1	Model 2	Model 3	Model 4
Total effect of LQR	1.21 [1.17, 1.25]	1.19 [1.15, 1.23]	1.17 [1.13, 1.21]	1.14 [1.10,1.18]
Indirect effects of LQR				
Combined indirect effect	1.17 [1.14, 1.21]	1.12 [1.09, 1.15]	1.11 [1.08, 1.14]	1.10 [1.07, 1.13]
Specific indirect effect via initial cognitive status	1.11 [1.08, 1.14]	1.07 [1.05, 1.09]	1.06 [1.04, 1.08]	1.05 [1.03, 1.07]
Specific indirect effect via rate of cognitive change	1.05 [1.03, 1.08]	1.04 [1.02, 1.07]	1.04 [1.02, 1.08]	1.04 [1.02, 1.07]
Direct effect of LQR	1.05 [1.00, 1.10]	1.08 [1.03, 1.14]	1.07 [1.01, 1.13]	1.05 [1.01, 1.11]

*Notes:* LQR = low-quality responding. Values in square brackets are 99% confidence intervals.

### Robustness of Effects Across Individual LQR Indicators

Finally, we repeated the analytic steps for each of the five individual LQR indicator variables to evaluate the robustness of the results across different indicators in sensitivity analyses. We found that the results were consistent for four of the indicators, namely, measures of response inconsistency, multivariate outliers, misfitting item response patters, and item nonresponses. In the models with all covariates, higher values on all of the individual LQR indicators were significantly associated with concurrently lower initial cognitive status, and with steeper cognitive decline in subsequent years. All indicators were significantly associated with a greater mortality risk, with significant indirect effects via initial cognitive status and rates of cognitive change. The exception was the LQR indicator of acquiescent responding, for which the results only partially confirmed the hypotheses. Specifically, in the final models, greater acquiescent responding was concurrently associated with lower initial cognitive functioning scores, but was not associated with subsequent rates of cognitive change or mortality risk (see Supplementary Material).

## Discussion and Implications

The extent to which individuals provide inattentive, random, aberrant, or low-quality responses in questionnaires has received increasing attention among psychologists in recent years. Whereas LQR is generally treated as a methodological nuisance that threatens the reliability and validity of self-report data, this study demonstrates that LQR represents a substantive indicator predicting cognitive decline and mortality risk at older ages. Consistent with prior studies (Kutschar et al., 2019; Lechner & Rammstedt, 2015), we found that LQR was cross-sectionally associated with lower cognitive abilities in the sample of older adults. Expanding on prior research, our results showed that LQR at baseline predicted subsequent cognitive decline and mortality over the 10 years of follow-up. Even though the standardized effect sizes relating LQR with participants’ cognitive trajectories were small, the prospective associations remained after controlling for known risk factors of cognitive decline and mortality. These results are in line with the idea that LQR might be an early behavioral indicator of subtle performance deficits that precede functioning declines late in life. However, our results should not be mistaken to suggest causality, and alternative explanations for the observed relationships are also possible. LQR, together with other behavior manifestations such as the inability to manage finances and difficulties with instrumental activities of daily living, might be a reflection and consequence of underlying cognitive deficits or might temporally codevelop with cognitive declines. Worse general health trajectories may also commonly underlie increases in LQR, cognitive declines, and mortality risk.

Reduced cognitive functioning and cognitive decline have previously been shown to predict mortality in older adults, controlling for biomedical risk factors (Batterham et al., 2012). We found that participants’ baseline cognitive status and rate of cognitive decline together accounted for about two thirds of the total effect of LQR on mortality risk in indirect effects models. This suggests that in addition to its relationships with cognitive functioning, LQR might be associated with additional behavioral, physiological, and psychological risk factors relevant for longevity, such as an increased acceleration of physical, social, and mental health deficits. We encourage the field to more fully explore the role of these different pathways that may link LQR to mortality. A more precise understanding of these pathways may also be supported by distinguishing among causes of death.

Consistent with previous studies (Bowling et al., 2016; Huang et al., 2015; Schneider et al., 2018), we found that the various indicators of LQR generally converged with one another. Our summary index of LQR had high internal consistency (with a Cronbach alpha exceeding .80), and sensitivity analyses showed that four of the five indicators gave very similar results. These findings show that LQR can be effectively measured with several indicators that can be derived from self-report data patterns. The fifth indicator, acquiescent responding, was not significantly associated with cognitive change or mortality. Even though prior research has found relationships between acquiescent responding and lower cognitive abilities among older adults (Lechner & Rammstedt, 2015; Schneider, 2018), acquiescent response tendencies have generally been viewed as reflecting enduring individual differences in personality, culture, and cognitive style (Van Vaerenbergh & Thomas, 2013), which may only be weakly related to cognitive changes and mortality risk.

Even though past survey research has acknowledged the role of lower cognitive abilities as a cause or correlate of LQR (Conijn et al., 2020; Krosnick, 1991), most commonly, problems with LQR in surveys have been attributed to a lack of respondent motivation, as reflected in labels such as “careless” and “insufficient effort” responding that predominate in research on these response behaviors. A particular focus has been on volitional aspects of low motivation as a source of LQR in younger study populations; for example, participants in student samples who may not be willing or interested in investing effort when responding to a survey for course credits (Meade & Craig, 2012). However, there are other aspects of motivation that might drive LQR and that are potentially more indicative of functioning problems in older adults, such as problems with executive and self-control functions that can make it difficult to focus, follow instructions, inhibit competing responses, and maintain attentional focus on relevant stimuli over extended periods amidst potential environmental distractions (Ferguson et al., 2021). To gain a better understanding of the role of LQR as a substantive variable in aging research, it will be important to disentangle the cognitive-motivational underpinnings of LQR, including the extent to which different LQR indicators are sensitive to specific aspects of motivation and cognition.

This study has several strengths including the use of a large U.S. national study sample of older men and women, a prospective follow-up period of up to 10 years, and the use of multiple previously validated indicators of LQR. Importantly we were able to adjust our latent growth curve models for death, enabling us to obtain more accurate estimates of cognitive change. Several study limitations should also be noted. One weakness of this study is the potential of selection bias, as HRS participants who did not return the leave-behind questionnaire differed on several demographic variables (age, education, race, marital status) from those who entered the current analyses, and they were also more likely to die over the follow-up period. We analyzed LQR among participants living in the United States, and the results may not generalize to other countries. The analyses were limited to a single, albeit large, data set, and only all-cause mortality was available to us for the present analyses. Relatedly, even though we used objective cognitive test scores in our analyses, we cannot say whether specific cognitive functioning domains are more or less closely related to LQR. Given that completing a questionnaire is a complex cognitive task with multiple components that include question interpretation, retrieval and integration of relevant information from memory, and adapting responses to changing answer formats (Krosnick, 1991; Tourangeau et al., 2000), we speculate that LQR may be broadly related to multiple cognitive processes and domains. Furthermore, the survey responses analyzed were limited to paper-and-pencil assessments, and the results may not generalize to other survey modalities.

Although we focused on LQR as a marker of cognitive decline and mortality, it is important to reiterate that LQR adversely affects data quality. Because of this, prior research has recommended that participants who strongly engage in LQR should not be included in the analyses (Huang et al., 2015; Johnson, 2005; Meade & Craig, 2012; Schneider et al., 2018). While the impact of LQR on reductions in data quality certainly is a valid concern, one needs to be mindful of such a decision as it can risk the exclusion of a meaningful subset of participants and limit the representativeness of the sample (Bowling et al., 2016). Especially for older samples, it may be important to consider strategies to improve the quality of their self-report data. Such strategies could include careful consideration of the self-report assessment format. For example, it is possible that face-to-face interview formats might be preferable over self-administered self-report (including paper-pencil and web-based) questionnaires for respondents who might be willing but not fully able to provide accurate self-reports. The presence of an interviewer could facilitate question comprehension and increase participant motivation (Meade & Craig, 2012). Interestingly, this might have the dual effect of increasing data quality and simultaneously reducing the ability of LQR to serve as a marker of health outcomes. At the same time, face-to-face interviews might not be practical in all research contexts and may increase the likelihood of socially desirable responding (Heerwegh, 2009). Prior research has argued that social desirability is especially important to consider in research with older adults because of their increased tendency to present themselves favorably to counter common aging stereotypes of mental and physical deterioration (Fastame & Penna, 2012). We encourage future research to examine LQR with different modes of assessments and explore effective ways to reduce it.

In sum, our study demonstrates that LQR can predict cognitive decline and mortality risk at older ages. As such, our results bolster the scientific argument that LQR should not be exclusively viewed as a data quality issue but instead as conceptually and practically meaningful individual difference marker. Self-report questionnaires are not formally designed as cognitive tasks but this study shows that LQR indicators derived from self-report measures provide objective, performance-based information about individuals’ functioning and survival. Thus, examining LQR in survey responses may facilitate the development of new strategies for early detection of pathological aging. Self-report surveys are ubiquitous in social science, and indicators of LQR may be of broad relevance as predictors of cognitive and health trajectories at older ages.

## Supplementary Material

igac027_suppl_Supplementary_Appendix
