# Supplementary material for: Quality of Survey Responses at Older Ages Predicts Cognitive Decline and Mortality Risk
Source: Innov Aging. 2022 Apr 20;6(3):igac027. doi: 10.1093/geroni/igac027 (PMC9155162; doi:10.1093/geroni/igac027)
Supplement: igac027_suppl_Supplementary_Appendix [file igac027_suppl_supplementary_appendix.docx]

**Quality of survey responses at older ages predicts cognitive decline and mortality risk**

**-- Online supplement --**

Index

Table S1 summarizes the psychosocial scales used to derive indices of low-quality responding (page 2).

Table S2 to Table S11 show the study results based on individual indicators of low-quality responding (pages 3-7).

The final section provides details on the computation of the indicators of low-quality responding (pages 8-12).

Table S1: Psychosocial scales in the “leave-behind” questionnaire package used to derive indices of low-quality responding

| Scale | N items | Response scale | Cronbach alpha^b^ |
| --- | --- | --- | --- |
| Life satisfaction | 5 | 6/7-point (strongly disagree – strongly agree)^a^ | .87 |
| Cynical hostility | 5 | 6-point (strongly disagree – strongly agree) | .78 |
| Optimism | 6 | 6-point (strongly disagree – strongly agree) | .71 |
| Hopelessness | 4 | 6-point (strongly disagree – strongly agree) | .85 |
| Loneliness | 3 | 3-point (often – hardly ever or never) | .81 |
| Neighborhood physical disorder | 4 | 7-point [positive vs negative descriptors as scale anchors, specific for each item] | .74 |
| Neighborhood social cohesion | 4 | 7-point [positive vs negative descriptors as scale anchors, specific for each item] | .85 |
| Constraints on personal control | 5 | 6-point (strongly disagree – strongly agree) | .85 |
| Perceived mastery | 5 | 6-point (strongly disagree – strongly agree) | .89 |
| Religiosity/Spirituality | 4 | 6-point (strongly disagree – strongly agree) | .92 |
| Everyday discrimination | 5 | 6-point (almost every day – never) | .80 |
| Social effort/reward balance | 3 | 5-point (strongly disagree – strongly agree) | .79 |
| Extraversion | 5 | 4-point (a lot – not at all) | .74 |
| Agreeableness | 5 | 4-point (a lot – not at all) | .78 |
| Neuroticism | 4 | 4-point (a lot – not at all) | .70 |
| Conscientiousness ^c^ | 4 | 4-point (a lot – not at all) | .69 |
| Openness to experience | 7 | 4-point (a lot – not at all) | .78 |
| Purpose in life | 7 | 6-point (strongly disagree – strongly agree) | .73 |
| Anxiety | 5 | 4-point (never – most of the time) | .81 |
| Anger-in | 4 | 4-point (almost never – almost always) | .77 |
| Anger-out | 7 | 4-point (almost never – almost always) | .87 |

*Note*: For details on each questionnaire and item wording, see Smith J, Fisher GG, Ryan L, Clarke P, House J, Weir D. *Health and Retirement Study Psychosocial and Lifestyle Questionnaire 2006 - 2010: Documentation Report.* Ann Arbor, MI: University of Michigan; 2013.

^a^ Life satisfaction was administered using a 6-point scale in 2006 and a 7-point scale in 2008.

^b^ Chronbach alphas are for the current analysis sample.

^c^ One conscientiousness item with low item-total correlation was removed from the analyses.

**Results based on individual indicators of low-quality responding**

In each table, Model 1 presents results adjusted for age. Model 2 adjusted for sociodemographic covariates (age, gender, race, marital status, education), Model 3 additionally adjusted for physical health variables (health conditions, smoking status, physical activity), and Model 4 additionally adjusted for depressive symptoms

Table S2. *Response inconsistency* index as predictor of initial cognitive status and rates of cognitive change in latent growth models

|  | Model 1 | Model 2 | Model 3 | Model 4 |
| --- | --- | --- | --- | --- |
| Unstandardized coefficients |  |  |  |  |
| Effect on initial   cognitive status | -1.38  [-1.50,-1.27] | -0.70  [-0.80,-0.59] | -0.66  [-0.77,-0.55] | -0.56  [-0.67,-0.45] |
| Effect on yearly rate of   cognitive change | -0.04  [-0.06,-0.03] | -0.04  [-0.05,-0.02] | -0.03  [-0.05,-0.02] | -0.03  [-0.05,-0.01] |
| Standardized coefficients |  |  |  |  |
| Effect on initial   cognitive status | -0.33 [-0.36,-0.30] | -0.17 [-0.19,-0.14] | -0.16 [-0.18,-0.13] | -0.13 [-0.16,-0.11] |
| Effect on yearly rate of   cognitive change | -0.11 [-0.15,-0.07] | -0.10 [-0.14,-0.05] | -0.09 [-0.13,-0.04] | -0.08 [-0.13,-0.03] |

*Note*: Values in square brackets are 99% confidence intervals.

Table S3. Hazard ratios for total, indirect and direct effects of *response inconsistency* index on mortality

|  | Model 1 | Model 2 | Model 3 | Model 4 |
| --- | --- | --- | --- | --- |
| Total effect of LQR | 1.24 [1.18, 1.29] | 1.21  [1.15, 1.27] | 1.18  [1.12, 1.23] | 1.14  [1.08,1.19] |
| Indirect effects of LQR |  |  |  |  |
| Combined indirect effect | 1.16  [1.12, 1.19] | 1.11  [1.08, 1.13] | 1.10  [1.07, 1.13] | 1.08  [1.06, 1.11] |
| Specific indirect effect  via initial cognitive status | 1.10  [1.07, 1.12] | 1.06  [1.04, 1.08] | 1.05  [1.03, 1.07] | 1.04  [1.02, 1.06] |
| Specific indirect effect  via rate of cognitive change | 1.05  [1.03, 1.08] | 1.04  [1.02, 1.07] | 1.04  [1.02, 1.07] | 1.04  [1.01, 1.07] |
| Direct effect of LQR | 1.08  [1.03, 1.14] | 1.11  [1.05, 1.17] | 1.08  [1.03, 1.14] | 1.06  [1.001, 1.12] |

*Note*: Values in square brackets are 99% confidence intervals.

Table S4. *Multivariate outlier* index as predictor of initial cognitive status and rates of cognitive change in latent growth models

|  | Model 1 | Model 2 | Model 3 | Model 4 |
| --- | --- | --- | --- | --- |
| Unstandardized coefficients |  |  |  |  |
| Effect on initial   cognitive status | -1.54  [-1.66,-1.43] | -0.84  [-0.95,-0.74] | -0.81  [-0.92,-0.70] | -0.71  [-0.82,-0.60] |
| Effect on yearly rate of   cognitive change | -0.04  [-0.05,-0.02] | -0.03  [-0.05,-0.02] | -0.03  [-0.05,-0.01] | -0.03  [-0.04,-0.01] |
| Standardized coefficients |  |  |  |  |
| Effect on initial   cognitive status | -0.37 [-0.39,-0.34] | -0.20 [-0.23,-0.18] | -0.19 [-0.22,-0.17] | -0.17 [-0.20,-0.14] |
| Effect on yearly rate of   cognitive change | -0.10 [-0.15,-0.06] | -0.09 [-0.13,-0.04] | -0.08 [-0.13,-0.03] | -0.07 [-0.12,-0.02] |

*Note*: Values in square brackets are 99% confidence intervals.

Table S5. Hazard ratios for total, indirect and direct effects of *multivariate outlier* index on mortality

|  | Model 1 | Model 2 | Model 3 | Model 4 |
| --- | --- | --- | --- | --- |
| Total effect of LQR | 1.31 [1.25, 1.37] | 1.27  [1.21, 1.34] | 1.23  [1.17, 1.29] | 1.18  [1.12,1.24] |
| Indirect effects of LQR |  |  |  |  |
| Combined indirect effect | 1.15  [1.12, 1.19] | 1.11  [1.08, 1.14] | 1.10  [1.07, 1.13] | 1.09  [1.06, 1.12] |
| Specific indirect effect  via initial cognitive status | 1.10  [1.07, 1.13] | 1.07  [1.05, 1.09] | 1.06  [1.04, 1.08] | 1.05  [1.03, 1.07] |
| Specific indirect effect  via rate of cognitive change | 1.05  [1.02, 1.08] | 1.04  [1.01, 1.07] | 1.04  [1.01, 1.07] | 1.04  [1.01, 1.07] |
| Direct effect of LQR | 1.15  [1.08, 1.21] | 1.16  [1.10, 1.22] | 1.12  [1.06, 1.19] | 1.10  [1.03, 1.16] |

*Note*: Values in square brackets are 99% confidence intervals.

Table S6. *Misfitting item response pattern* index as predictor of initial cognitive status and rates of cognitive change in latent growth models

|  | Model 1 | Model 2 | Model 3 | Model 4 |
| --- | --- | --- | --- | --- |
| Unstandardized coefficients |  |  |  |  |
| Effect on initial   cognitive status | -1.39  [-1.50,-1.27] | -0.71  [-0.82,-0.61] | -0.70  [-0.81,-0.59] | -0.63  [-0.74,-0.52] |
| Effect on yearly rate of   cognitive change | -0.04  [-0.05,-0.02] | -0.03  [-0.05,-0.01] | -0.03  [-0.05,-0.01] | -0.03  [-0.04,-0.01] |
| Standardized coefficients |  |  |  |  |
| Effect on initial   cognitive status | -0.33 [-0.36,-0.30] | -0.17 [-0.20,-0.14] | -0.17 [-0.19,-0.14] | -0.15 [-0.18,-0.12] |
| Effect on yearly rate of   cognitive change | -0.09 [-0.14,-0.05] | -0.08 [-0.12,-0.03] | -0.07 [-0.12,-0.03] | -0.07 [-0.12,-0.02] |

*Note*: Values in square brackets are 99% confidence intervals.

Table S7. Hazard ratios for total, indirect and direct effects of *misfitting item response pattern* index on mortality

|  | Model 1 | Model 2 | Model 3 | Model 4 |
| --- | --- | --- | --- | --- |
| Total effect of LQR | 1.18 [1.13, 1.24] | 1.15  [1.10, 1.21] | 1.15  [1.09, 1.20] | 1.12  [1.07,1.17] |
| Indirect effects of LQR |  |  |  |  |
| Combined indirect effect | 1.15  [1.12, 1.18] | 1.11  [1.07, 1.13] | 1.09  [1.06, 1.12] | 1.08  [1.05, 1.11] |
| Specific indirect effect  via initial cognitive status | 1.10  [1.08, 1.13] | 1.06  [1.04, 1.08] | 1.05  [1.04, 1.07] | 1.05  [1.03, 1.06] |
| Specific indirect effect  via rate of cognitive change | 1.05  [1.02, 1.07] | 1.04  [1.01, 1.06] | 1.04  [1.01, 1.06] | 1.04  [1.01, 1.06] |
| Direct effect of LQR | 1.04  [0.99, 1.09] | 1.06  [1.01, 1.11] | 1.05  [1.00, 1.11] | 1.04  [0.99, 1.10] |

*Note*: Values in square brackets are 99% confidence intervals.

Table S8. *Acquiescent responding* index as predictor of initial cognitive status and rates of cognitive change in latent growth models

|  | Model 1 | Model 2 | Model 3 | Model 4 |
| --- | --- | --- | --- | --- |
| Unstandardized coefficients |  |  |  |  |
| Effect on initial   cognitive status | -0.51  [-0.63,-0.39] | -0.15  [-0.26,-0.05] | -0.17  [-0.28,-0.10] | -0.19  [-0.29,-0.08] |
| Effect on yearly rate of   cognitive change | -0.01  [-0.02,0.01] | -0.00  [-0.02,0.02] | -0.00  [-0.02,0.01] | -0.01  [-0.02,0.01] |
| Standardized coefficients |  |  |  |  |
| Effect on initial   cognitive status | -0.12 [-0.15,-0.10] | -0.04 [-0.06,-0.01] | -0.04 [-0.07,-0.02] | -0.04 [-0.07,-0.02] |
| Effect on yearly rate of   cognitive change | -0.02 [-0.07,0.02] | -0.01 [-0.05,0.04] | -0.01 [-0.06,0.04] | -0.01 [-0.06,0.03] |

*Note*: Values in square brackets are 99% confidence intervals.

Table S9. Hazard ratios for total, indirect and direct effects of *acquiescent responding* index on mortality

|  | Model 1 | Model 2 | Model 3 | Model 4 |
| --- | --- | --- | --- | --- |
| Total effect of LQR | 0.99 [0.95, 1.03] | 0.98  [0.94, 1.03] | 0.98  [0.97, 1.06] | 1.02  [0.97,1.06] |
| Indirect effects of LQR |  |  |  |  |
| Combined indirect effect | 1.05  [1.03, 1.07] | 1.02  [1.00, 1.04] | 1.02  [1.00, 1.04] | 1.02  [1.00, 1.04] |
| Specific indirect effect  via initial cognitive status | 1.04  [1.03, 1.05] | 1.01  [1.00, 1.02] | 1.01  [1.00, 1.02] | 1.01  [1.01, 1.02] |
| Specific indirect effect  via rate of cognitive change | 1.01  [0.99, 1.03] | 1.00  [0.98, 1.02] | 1.01  [0.98, 1.03] | 1.01  [0.98, 1.03] |
| Direct effect of LQR | 0.95  [0.90, 0.99] | 0.97  [0.92, 1.02] | 1.00  [0.95, 1.04] | 1.00  [0.95, 1.05] |

*Note*: Values in square brackets are 99% confidence intervals.

Table S10. *Item non-response* index as predictor of initial cognitive status and rates of cognitive change in latent growth models

|  | Model 1 | Model 2 | Model 3 | Model 4 |
| --- | --- | --- | --- | --- |
| Unstandardized coefficients |  |  |  |  |
| Effect on initial   cognitive status | -0.96  [-1.09,-0.84] | -0.49  [-0.60,-0.38] | -0.48  [-0.59,-0.37] | -0.45  [-0.56,-0.35] |
| Effect on yearly rate of   cognitive change | -0.03  [-0.05,-0.02] | -0.02  [-0.04,-0.01] | -0.02  [-0.04,-0.004] | -0.02  [-0.04,-0.003] |
| Standardized coefficients |  |  |  |  |
| Effect on initial   cognitive status | -0.23 [-0.26,-0.20] | -0.12 [-0.14,-0.10] | -0.11 [-0.14,-0.09] | -0.11 [-0.13,-0.08] |
| Effect on yearly rate of   cognitive change | -0.09 [-0.14,-0.04] | -0.06 [-0.12,-0.01] | -0.06 [-0.11,-0.01] | -0.06 [-0.11,-0.01] |

*Note*: Values in square brackets are 99% confidence intervals.

Table S11. Hazard ratios for total, indirect and direct effects of *item non-response* index on mortality

|  | Model 1 | Model 2 | Model 3 | Model 4 |
| --- | --- | --- | --- | --- |
| Total effect of LQR | 1.09 [1.05, 1.14] | 1.08  [1.04, 1.12] | 1.07  [1.02, 1.11] | 1.06  [1.02,1.11] |
| Indirect effects of LQR |  |  |  |  |
| Combined indirect effect | 1.12  [1.09, 1.15] | 1.07  [1.05, 1.11] | 1.07  [1.04, 1.10] | 1.07  [1.04, 1.09] |
| Specific indirect effect  via initial cognitive   status | 1.07  [1.05, 1.09] | 1.04  [1.03, 1.06] | 1.04  [1.02, 1.05] | 1.03  [1.02, 1.05] |
| Specific indirect effect  via rate of cognitive   change | 1.04  [1.02, 1.07] | 1.03  [1.004, 1.05] | 1.03  [1.003, 1.06] | 1.03  [1.003, 1.06] |
| Direct effect of LQR | 0.99  [0.95, 1.04] | 1.01  [0.97, 1.06] | 1.01  [0.96, 1.06] | 1.01  [0.96, 1.05] |

*Note*: Values in square brackets are 99% confidence intervals.

**Operationalization of indicators of low-quality responding derived from the leave behind questionnaires in the Health and Retirement Study**

Response inconsistency index

We calculated an index of response inconsistency by estimating the residual item-level variability around a person’s “true” scale scores using multilevel modeling. We first transformed all survey items onto a common 0-10 metric. Negatively worded items were reverse-coded as described in the Health and Retirement Study Psychosocial and Lifestyle Questionnaire Documentation Report (Smith et al., 2013). For each respondent, we then estimated a multilevel model that expresses each person’s responses on all 102 items using a pair of linked models: one at the item level (Level 1) and one at the scale level (Level 2), as follows:

| Level 1: *y_ij_* = δ_0_*_j_* + ε*_ij_*, where ε*_ij_* ~ *N*(0,σ^2^). | (1a) |
| --- | --- |
| Level 2: δ_0_*_j_* = γ_00_ + *u*_0_*_j_*, where *u*_0_*_j_* ~ *N*(0,τ^2^_00_). | (1b) |

At Level 1, the response y for item *i* on a given scale *j* is expressed as the sum of a “true” scale score (δ_0_*_j_*) and a random error (ε*_ij_*). At Level 2, the “true” scale scores are expressed as the sum of a grand mean scale score γ_00_ and scale-specific deviations around this grand mean *u*_0_*_j_*. τ_00_^2^ represents the latent variance of the “true” scale scores and σ^2^ represents the amount of residual variance (i.e., the amount of response inconsistency).

We estimated the multilevel models using the MIXED procedure in SAS version 9.4 (Cary, NC). Missing values were accommodated using full information maximum likelihood parameter estimation. We log transformed the values of the response inconsistency indicator variable to normalize its distribution for the analyses.

Multivariate outlier index

We used the Mahalanobis distance to calculate an index of multivariate outliers. The Mahalanobis distance is an extension of univariate (single-variable) outlier statistics to multivariate response vectors (i.e., multiple correlated variables). It expresses how many standard deviations a given respondent is away from the centroid (the midpoint or center of mass) of the multivariate distribution of scores. Formally, for a data matrix **X** containing *n* respondents *x_i_* measured by *p* items, the squared Mahalanobis distance (MD^2^) is the multivariate distance between respondent’s response vector and the vector of sample means $\bar{x}$, expressed as

| ${MD}^{2}=(x_{i}- \bar{x})C_{x}^{-1}(x_{i}- \bar{x})^{T}.$ | (2) |
| --- | --- |

*C_x_* is the variance-covariance matrix of the items, such that the Mahalanobis distance takes the correlated nature of survey items into account. For example, giving responses in opposite directions for two positively correlated variables (selecting a high value for one and low value for the other variable) would represent an “unusual” pattern of responses that would be more outlying.

We calculated the MD^2^ of each person’s response pattern across all 102 items using the REG procedure in SAS 9.4 (Cary, NC). Missing values were accommodated with multiple imputation, where MD^2^ values were calculated from and averaged over 20 multiply imputed datasets. We subsequently log transformed resulting MD^2^ value to normalize the distribution of the multivariate outlier index for the analyses.

Item response theory-based person-fit index

We calculated an item response theory (IRT) based index of *misfitting* item response patterns using the *l_z_* person-fit statistic. Person-fit statistics are a measure to quantify the extent to which a particular item score patterns is consistent or inconsistent with the expected pattern given an underlying IRT model. Using IRT it is possible to estimate the likelihood that a respondent with a given level on the latent “trait” (e.g., with a certain level of optimism, conscientiousness, life satisfaction) selects a particular combination of response options across the items of a scale. When this likelihood is low, the person’s pattern of scores on the scale is deemed inconsistent. For simplicity, consider a scale with 3 items with three ordinal response options (1, 2, and 3) per item. Assume that for a specific level on the latent trait, the IRT model predicts that a person should choose the 3 response options with the following probabilities: item A (0.3, 0.6 and 0.1), item B (0.7, 0.2, and 0.1), item C (0.2, 0.5, and 0.3). Then the pattern of selected responses (2, 1, 2) is the most likely and the pattern (3, 3, 1) is the least likely for this person. The *l_z_* person-fit statistic is defined as the standardized log-likelihood of a pattern of items scores under an IRT model (there are several different IRT models, and we used the graded response model as one of the most common models). The specific computation of the *l_z_* person-fit statistic is described in Drasgow, Levine, and Williams (1985).

We used the R software package PerFit (Tendeiro, Meijer, & Niessen, 2016) to calculate the *l*_z_ index, separately for each of the scales of the leave behind questionnaire package. The package handles missing values via Hotdeck imputation. We log-transformed the scores of the *l*_z_ index to normalize their distributions (a constant of 0.1 was added to allow log transformation of zero values), and subsequently averaged the resulting scores across scales for each respondent.

Acquiescent responses

We calculated an index of acquiescent responding, defined as agreeing to statements or giving affirmative (positive) responses regardless of the content of questions (Knowles & Condon, 1999). Following recommended psychometric procedures, we used a two-factorial nominal response model (NRM) to capture acquiescent responding (Falk & Cai, 2016; Schneider, 2018). This model addresses limitations of sum-score based methods that merely count the number of times that participants selected an affirmative response option, which confounds the measurement of acquiescence and the measurement of substantive traits. The NRM addresses this issue by separating substantive and acquiescent response tendencies into two latent factors (Falk & Ju, 2020; Schneider, 2018).

In the NRM, the probability of a participant selecting response option *x* on item *i* can be expressed as

| $P_{i(x)}= \frac{exp({c_{i\left( x \right)}+a}_{i\left( x \right)}\theta)}{\sum_{k=1}^{K} exp(c_{i\left( k \right)}+a_{i(k)}\theta)},$ | (3) |
| --- | --- |

where *P_i(x)_* represents the probability of choosing response option *x* for item *i*, *k* indicates the elements of a vector of *K* response options (with *k* = 1, . . ., *K*), and θ is a latent factor underlying the participant’s responses, *c_i(x)_* denotes an intercept for response option *k* and *a_i(x)_* is a category slope for the relationship between response option *k* and the latent variable.

The two-factorial NRM simultaneously estimates 2 latent factors corresponding to (1) a substantive construct (e.g., depression, optimism, etc.) and (2) acquiescent responding, by constraining the item category slopes (*a* parameters) of each factor. The *a* parameters of the factor capturing the substantive construct were constrained so that choosing higher response options corresponds with higher levels on the latent variable. For example, for Likert scale items with 5 response categories (1=Strongly disagree, 2=Slightly disagree, 3=Neither agree nor disagree, 4=Slightly agree, 5=Strongly agree), the category slopes are defined as [0 1 2 3 4] for items expressing higher depression (or higher optimism, etc.), and reverse coded for items expressing lower depression (or lower optimism, etc.). A second factor capturing acquiescent responding was estimated by constraining the *a* parameters for the second factor to [0 0 0 0 1], such that a higher likelihood of “strongly agree” responses corresponds with higher levels on the acquiescent response factor.

The two-factorial NRM was estimated separately for each of the scales in the leave behind questionnaire package using M*plus* version 8.5 (Muthén & Muthén, 2017). Details on M*plus* code to estimate the model have been described previously (Falk & Ju, 2020; Schneider, 2018). Missing values were accommodated with full information maximum likelihood parameter estimation. We obtained the factor scores on the acquiescent response factor for each scale, and then averaged them within each respondent for subsequent data analysis.

Item non-response

We obtained an index of item non-response by calculating the proportion of skipped items (i.e., missing values) across the102 items contained in the 21 scales of the leave behind questionnaire package, as follows:

| *P*_miss_ = N_miss_/102. | (4) |
| --- | --- |

We arcsine transformed the values of the index to stabilize its variance across respondents for the analyses.

References

Drasgow, F., Levine, M. V., & Williams, E. A. (1985). Appropriateness measurement with polychotomous item response models and standardized indices. *British journal of mathematical and statistical psychology, 38*(1), 67-86.

Falk, C. F., & Cai, L. (2016). A flexible full-information approach to the modeling of response styles. *Psychological Methods, 21*(3), 328-347.

Falk, C. F., & Ju, U. (2020). Estimation of response styles using the multidimensional nominal response model: A tutorial and comparison with sum scores. *Frontiers in psychology, 11*, 72.

Knowles, E. S., & Condon, C. A. (1999). Why people say" yes": A dual-process theory of acquiescence. *Journal of Personality and Social Psychology, 77*(2), 379-386.

Muthén, L. K., & Muthén, B. O. (2017). *Mplus: Statistical Analysis with Latent Variables: User's Guide (Version 8).* Los Angeles, CA: Muthén & Muthén.

Schneider, S. (2018). Extracting Response Style Bias From Measures of Positive and Negative Affect in Aging Research. *The Journals of Gerontology Series B: Psychological Sciences and Social Sciences, 73*, 64-74. doi:10.1093/geronb/gbw103

Smith, J., Fisher, G. G., Ryan, L., Clarke, P., House, J., & Weir, D. (2013). *Health and Retirement Study Psychosocial and Lifestyle Questionnaire 2006 - 2010: Documentation Report*. Ann Arbor, MI: University of Michigan.

Tendeiro, J. N., Meijer, R. R., & Niessen, A. S. M. (2016). PerFit: An R package for person-fit analysis in IRT. *Journal of Statistical Software, 74*(5), 1-27.
